# Elucidation of Organohalogenochromism (OHC) of D–A Pyridinium and D‐π‐A Pyridinium Dyes: Effect of Halogen Bond

**DOI:** 10.1002/asia.202500746

**Published:** 2025-06-23

**Authors:** Kumpei Kozuka, Keiichi Imato, Yousuke Ooyama

**Affiliations:** ^1^ Applied Chemistry Program Graduate School of Advanced Science and Engineering Hiroshima University 1‐4‐1 Kagamiyama, Higashi Hiroshima 739‐8527 Japan

**Keywords:** Halogen bond, Intramolecular charge transfer, Organohalogenochromism, Positive surface electrostatic potential, Pyridinium

## Abstract

Bathochromic shift‐type OHC (b‐OHC) was found in donor–acceptor (D–A) type pyridinium dye bearing halide ion as a counter anion, as well as donor‐π‐acceptor (D‐π‐A) type pyridinium dye; the intramolecular charge transfer‐based photoabsorption maxima (*λ*_max_^abs^) in halogenated solvents show a large bathochromic shift, in comparison with those in non‐halogenated solvents. It was revealed that there is a good relationship between the most positive surface electrostatic potential (*V*_S,max_) values associated with the most positive *σ*‐hole on halogen atoms in organohalogen molecule and the *λ*_max_^abs^, indicating that the b‐OHC of the D–A and D‐π‐A pyridinium dyes is attributed to the formation of halogen bond (XB) or complex such as [R─X·Y]^−^ between the halogen atom (X) of organohalogen molecule and the counter anion (Y) of dye molecule. Moreover, the plots of the *λ*_max_^abs^ against the *V*_S,max_ values demonstrated that the b‐OHC characteristic of D–A pyridinium dye is lower than that of the D‐π‐A pyridinium dye. It was suggested that the formation of XB induces a decrease in the ring current of the pyridinium ring, that the LUMO is mainly localized, resulting in the expression of b‐OHC. Consequently, this work offers a deeper insight into the mechanism for the expression and the origin of OHC.

## Graphical abstract

Bathochromic shift‐type OHC (b‐OHC) was found in donor–acceptor (D–A) type pyridinium dye bearing halide counter anion, as well as donor‐π‐acceptor (D‐π‐A) type pyridinium dye. This work revealed that formation of halogen bond (XB) or complex such as [R─X·Y]^−^ between halogen atom (X) of organohalogen molecule and counter anion (Y^−^) of dye molecule contributes to expression of b‐OHC.

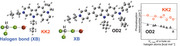

## 1 Introduction

In halogenated solvents, including dichloromethane and dibromomethane, some organic dyes exhibit a significant hypsochromic or bathochromic shift of photoabsorption band that has long been noticed by many researchers.^[^ ^1^, ^2^, ^3^, ^4^, ^5^, ^6^, ^7^, ^8^, ^9^, ^10^, ^11^, ^12^, ^13^, ^14^, ^15^, ^16^, ^17^, ^18^, ^19^, ^20^, ^21^, ^22^, ^23^, ^24^, ^25^, ^26^, ^27^, ^28^, ^29^, ^30^, ^31^, ^32^, ^33^, ^34^ ^]^ Nevertheless, such specific solvatochromism has only recently been termed organohalogenochromism (OHC),^[^ ^1^, ^6^ ^]^ and thus it is recognized as a photophysical phenomenon that is totally different from a common solvatochromism depending on solvent polarity parameter (*E*_T_(30)), normalized solvent polarity value (*E*_T_^N^) or dielectric constant (*ε*_r_) of solvent.^[^ ^35^, ^36^, ^37^, ^38^, ^39^, ^40^, ^41^ ^]^ So far, bathochromic shift‐type OHC (b‐OHC) has been found in a certain donor‐π‐acceptor (D‐π‐A) type cationic dyes bearing a counter anion, which are composed of a strong electron‐donating (D) moiety (e.g., dialkyl or diaryl amino group) and a strong electron‐withdrawing (A) cationic moiety (e.g., pyridinium or benzothiazolium ring) linked by a π‐conjugated bridge, so that they exhibit an intense photoabsorption band based on the intramolecular charge transfer (ICT) from the D to the A moiety.^[^ ^1^, ^2^, ^3^, ^4^, ^5^, ^7^, ^8^, ^9^, ^10^, ^11^, ^12^, ^13^, ^14^, ^15^, ^16^, ^17^, ^18^, ^19^, ^20^ ^]^ However, the research on OHC remains in a phenomenological investigation, although organohalogenochromic dyes have a great potential for developing optical sensing system for visualization and detection of toxic volatile organohalogen compounds (VOHCs). Therefore, in order to provide a direction in the molecular design for expressing OHC reliably and to create functional dye materials for colorimetric detection of VOHCs, further fundamental studies to lead to comprehensive elucidation of the origin of OHC are absolutely required.

**Figure 1:**
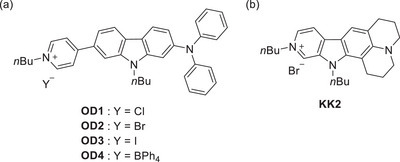
Chemical structures of (a) D‐π‐A type pyridinium dyes **OD1**‐**4** in previous work and (b) D–A type pyridinium dye **KK2** in this work.

Meanwhile, we have reported that D‐π‐A type pyridinium dyes **OD1**‐**4** bearing various counter anions (Y^−^ = Cl^−^, Br^−^, I^−^, or BPh_4_^−^), which have a diphenylamino group as a D moiety and a pyridinium ring as an A moiety connected by a carbazole skeleton as a π‐conjugated bridge, show a significant bathochromic shift of ICT‐based photoabsorption maximum wavelengths (*λ*_max_^abs^) in halogenated solvents, compared with those in non‐halogenated solvents, indicating a pronounced b‐OHC (Figure 1a).^[^ ^2^ ^]^ More recently, in our continuous efforts to elucidate the mechanism for expression of b‐OHC, we found that the b‐OHC of the D‐π‐A pyridinium dyes may be attributed to the formation of halogen bond (XB) between the halogen atom of the organohalogen molecule and the counter anion of the dye molecule. The XB, which is depicted as R─X···Y, is an electrostatic interaction between the region (*σ*‐hole) with the positive surface electrostatic potential at the extremity of a halogen atom (X) in a molecular entity (R─X) as XB donor and a lone‐pair‐possessing atom, π‐system, or anion (Y) in an another electron‐rich molecular entity as XB acceptor.^[^ ^42^, ^43^, ^44^, ^45^, ^46^, ^47^, ^48^, ^49^, ^50^ ^]^

Thus, in this work, in order to gain a deeper insight into the origin of OHC with the objective of XB between the organohalogen and the dye molecules, we have designed and prepared the D–A type pyridinium dye **KK2** bearing bromide ion (Br^−^) as a counter anion which has julolidine part as the D moiety (Figure 1b). It is expected that the difference in the electronic structure between **OD2** and **KK2** affects the magnitude of OHC characteristics. Herein, based on the ^1^H NMR spectroscopies, cyclic voltammetry (CV), the single‐crystal X‐ray structural analyses, and the theoretical investigations using density functional theory (DFT) calculation, we offer the mechanism for the expression of OHC by the influence of XB on the electronic structure of the dye molecule.

## 2 Results and Discussion

**Figure 2:**
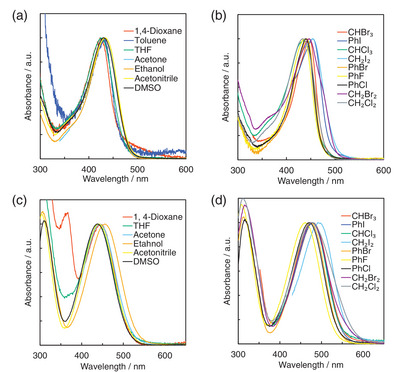
Photoabsorption spectra of (a) **KK2** and (c) **OD2** in non‐halogenated solvents and (b) **KK2** and (d) **OD2** in halogenated solvents.

The D–A type pyridinium dye **KK2** was prepared from the D–A type pyridine dye **ET‐1**.^[^ ^51^, ^52^ ^]^ and *n*‐butyl bromide (Scheme S1). **KK2** shows the photoabsorption maximum wavelength (*λ*_max_^abs^) at around 420–455 nm originating form ICT excitation from julolidine part as D moiety to a pyridinium ring as A moiety both in halogenated and non‐halogenated solvents (Figure 2 and Table 1), and thus the ICT‐based *λ*_max_^abs^ of **KK2** appear in a shorter wavelength region compared to those (ca. 440–495 nm) of **OD2**. Meanwhile, the molar extinction coefficient values (*ε*_max_ = ca. 8,500–23,500 M^−1^ cm^−1^) of *λ*_max_^abs^ for **KK2** are smaller than those (*ε*_max_ = ca. 16,000–40,500 M^−1^ cm^−1^) for **OD2**.

**Table 1:** Photophysical data of KK2 and OD2 in various solvents.

		*E*_T_(30)^a^ ^)^				*V*_S,max_ ^e^ ^)^	KK2	OD2
No.	Solvent	(kcal mol^−1^)	*f*(*n* ^2^)^b^ ^)^	*Δf* ^c^ ^)^	*ε*_r_ ^d^ ^)^	(kcal mol^−1^)	$\lambda_{\max}^{\mathrm{abs}}$ (*ε*_max_/M^−1^ cm^−1^)	$\lambda_{\max}^{\mathrm{abs}}$ (*ε*_max_/M^−1^ cm^−1^)
1	1,4‐Dioxane	36.0	0.202	0.022	2.22	–	423 (–^f^ ^)^)	442(–^f^ ^)^)
2	Toluene	33.9	0.215	0.025	2.38	–	429 (–^f^ ^)^)	–^f^ ^)^
3	CHBr_3_	37.7	0.260	0.087	4.40	20.46	446 (23,400)	479 (19,100)
4	PhI	36.2	0.260	0.092	4.59	17.85	454 (19,300)	480 (16,100)
5	CHCl_3_	39.1	0.210	0.149	4.81	15.86	440 (22,200)	477 (25,500)
6	CH_2_I_2_	36.5	0.286	0.085	5.32	22.34	453 (12,900)	494 (27,200)
7	PhBr	36.6	0.244	0.130	5.45	9.96	443 (8800)	472 (–^f^ ^)^)
8	PhF	37.0	0.218	0.157	5.47	–^g^ ^)^	436 (–^f^ ^)^)	463 (–^f^ ^)^)
9	PhCl	36.8	0.234	0.145	5.69	4.60	440 (10,700)	470 (–^f^ ^)^)
10	THF	37.4	0.197	0.210	7.52	–	425 (–^f^ ^)^)	440 (30,100)
11	CH_2_Br_2_	39.4	0.239	0.170	7.77	13.95	446 (19,600)	475 (31,300)
12	CH_2_Cl_2_	40.7	0.202	0.218	8.93	8.62	452 (16,800)	473 (32,600)
13	Acetone	42.2	0.179	0.286	21.0	–	434 (18,600)	443 (30,000)
14	Ethanol	51.9	0.181	0.290	25.3	–	434 (18,500)	455 (20,300)
15	Acetonitrile	45.6	0.174	0.306	36.6	–	432 (14,800)	440 (40,100)
16	DMSO	45.1	0.220	0.264	47.2	–	430 (16,300)	438 (21,900)

^a)^ Solvent polarity parameter (ref. [36, 37, 53]).

^b)^ Polarizability density (*n* ^2^–1/2*n* ^2 ^+ 1) (ref. [55]).

^c)^ Orientation polarizability (ref. [55]).

^d)^ Dielectric constant (ref. [55]).

^e)^ The most positive surface electrostatic potential (ref. [59, 60]).

^f)^ Poorly soluble.

^g)^ Negative value for fluorine atom.

**Figure 3:**
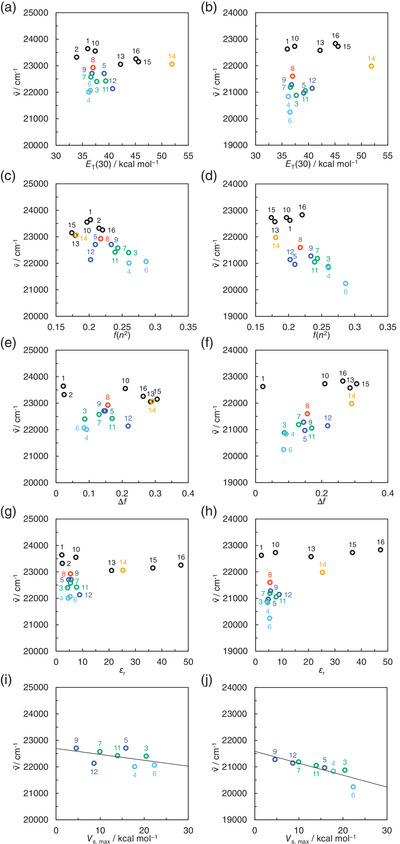
Plots of the photoabsorption maximum wavenumber (*v* ^∼^) of **KK2** against (a) solvent polarity parameter (*E*_T_(30)), (c) polarizability density (*n* ^2^–1/2*n* ^2 ^+ 1; *f(n* ^2^)), (e) orientation polarizability (*Δf*), (g) dielectric constant (*ε*_r_) of solvent, and (i) the most positive surface electrostatic potential (*V*_S,max_) associated with the most positive σ‐hole on halogen atoms in organohalogen solvent. Plots of the photoabsorption maximum wavenumber (*v* ^∼^) of **OD2** against (b) *E*_T_(30), (d) *f*(*n* ^2^), (f) *Δf*, (h) *ε*_r_ of solvent, and (j) *V*_S,max_. The numbers correspond to those of Table 1. The circles in black, red, blue, green, purple, light blue, and orange show non‐halogenated solvents, fluorobenzene, chlorinated solvents, brominated solvents, iodinated solvents, and ethanol, respectively.

In order to make clear the influence of solvent polarity on the ICT‐based *λ*_max_^abs^ of **KK2** and **OD2**, the wavenumbers (*v* ^∼^_max_^abs^) of the *λ*_max_^abs^ were plotted against solvent polarity parameter (*E*_T_(30)),^[^ ^36^, ^37^, ^53^ ^]^ polarizability density (*n* ^2^–1/2*n* ^2 ^+ 1; *f(n* ^2^)),^[^ ^54^, ^55^ ^]^ orientation polarizability (*Δf*),^[^ ^55^ ^]^ and dielectric constant (*ε*_r_) of solvent^[^ ^55^ ^]^ (Figure 3a–h). For both the dyes, these plots do not follow a linear relationship between them and remain on almost plateau among non‐halogenated solvents, indicating that the *v* ^∼^_max_^abs^ are nearly independent of solvent polarity. On the other hand, obviously, one can see that **KK2** and **OD2** show a significant bathochromic shift of *λ*_max_^abs^, that is, shifting of *v* ^∼^_max_^abs^ to lower wavenumber, in halogenated solvents compared with the *λ*_max_^abs^ (*v* ^∼^_max_^abs^) in non‐halogenated solvents; while the *ε*_r_ value (7.52) of THF is quite similar to that (7.77) of dibromomethane, the *λ*_max_^abs^ (446 nm and 475 nm) of **KK2** and **OD2** in dibromomethane appear at a longer wavelength region by 21 nm and 35 nm, respectively, than those (425 nm and 440 nm) in THF. It is worth mentioning here that in halogenated solvents the ICT‐based *λ*_max_^abs^ (ca. 435–455 nm) of **KK2** occur at a shorter wavelength region (a higher wavenumber range) compared to those (ca. 460–495 nm) of **OD2**. In the course of exploring the influences of halogenated solvents on the ICT‐based *λ*_max_^abs^ (*v* ^∼^_max_^abs^), we found that there is a good relationship between the most positive surface electrostatic potential (*V*_S,max_) values associated with the most positive *σ*‐hole on halogen atoms in organohalogen molecule and the *v* ^∼^_max_^abs^, indicating influence of the formation of halogen bond (XB) on the electronic structure of dye molecule. In general, the strength of XB regarding halogen atom (X) increases with an increase in the size of the halogen atom as Lewis acid, that is, in the order of F < Cl < Br < I. Indeed, the plots of the *v* ^∼^_max_^abs^ against the *V*_S,max_ values demonstrated that for both **KK2** and **OD2** the *v* ^∼^_max_^abs^ decrease linearly with the increase in the *V*_S,max_ values (Figure 3i,j). Consequently, the fact revealed that the b‐OHC of the D–A and D‐π‐A pyridinium dyes may be associated with the formation of XB between the halogen atom of organohalogen molecule and the counter anion of the dye molecule (Figure 3i,j). However, the correlation coefficient (*R* ^2^) value (0.235) and the slope (*m*_s_) value (−22.2) of the calibration curve for **KK2** are lower than those (0.733 and −45.1, respectively) for **OD2**, indicating that the b‐OHC characteristic of **KK2** is lower than that of **OD2**.

**Figure 4:**
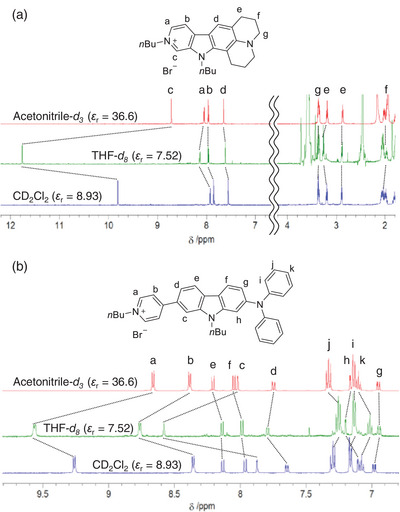
^1^H NMR spectra of (a) **KK2** and (b) **OD2** in acetonitrile‐*d*_3_, THF‐*d*_8_, and dichloromethane‐*d*_2_ (CD_2_Cl_2_).

Thus, in order to investigate the solvent effect on the electronic structure of the D–A and D‐π‐A pyridinium dyes, we performed the ^1^H NMR spectral measurements of **KK2** and **OD2** in acetonitrile‐*d*_3_ and THF‐*d*_8_ as non‐halogenated solvents and dichloromethane‐*d*_2_ as a halogenated solvent (Figure 4). One can see that there is a slight difference in the chemical shifts of the protons for D or D‐π moiety, that is, the julolidine moiety for **KK2** or the diphenylamino group and its nearby carbazole part for **OD2**, between the three deuterated solvents. On the other hand, significant differences in the chemical shifts of the A moiety were observed between halogenated and non‐halogenated solvents. For **KK2**, the signals of H_a_ and H_b_ of the pyridinium ring in dichloromethane‐*d*_2_ show upfield shifts compared to those in acetonitrile‐*d*_3_ and THF‐*d*_8_, while the signal for H_c_ of the pyridinium ring in dichloromethane‐*d*_2_ shows upfield and downfield shifts, respectively, compared to those in THF‐*d*_8_ and acetonitrile‐*d*_3_. Meanwhile, for **OD2** the signals of H_b_ of pyridinium ring, H_c_ and H_d_ of carbazole part near the pyridinium ring in dichloromethane‐*d*_2_ show upfield shifts compared to those in acetonitrile‐*d*_3_ and THF‐*d*_8_, while the signal for H_a_ of the pyridinium ring in dichloromethane‐*d*_2_ show upfield and downfield shift, respectively, compared to those in THF‐*d*_8_ and acetonitrile‐*d*_3_, as with the case of **KK2**. This result suggests a decrease in the ring current of the pyridinium and its nearby aromatic rings in halogenated solvents, compared to non‐halogenated solvents.^[^ ^56^ ^]^ Consequently, the fact offers the occurrence of intermolecular interaction between the organohalogen and the dye molecules, that is, the formation of XB between the halogen atom of organohalogen molecule and the counter anion of the dye molecule, leading to a decrease in the ring current of the pyridinium and its nearby aromatic rings in halogenated solvents.

**Figure 5:**
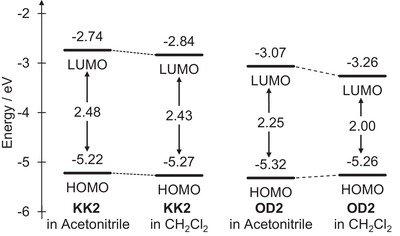
Energy level diagram for HOMO and LUMO of **KK2** and **OD2** estimated from cyclic voltammetry and photoabsorption spectral measurement.

Furthermore, in order to reveal the influence of the HOMO and LUMO energy levels of the D–A and D‐π‐A pyridinium dyes on the b‐OHC, we performed cyclic voltammetry (CV) in acetonitrile or dichloromethane containing 0.1 M tetrabutylammonium perchlorate (Bu_4_NClO_4_) to determine exactly the redox potential (Figure S5). The potentials were internally referenced to ferrocene/ferrocenium (Fc/Fc^+^). The cyclic voltammograms showed an irreversible oxidation wave at ca. 0.40‒0.50 V for **KK2** and ca. 0.45‒0.55 V for **OD2**, and in acetonitrile, the oxidation peak potential (*E*_pa_^ox^) for **KK2** is cathodically shifted by ca. 0.1 V, compared to that for **OD2**. Moreover, it was found that the *E*_pa_^ox^ for **KK2** and **OD2** in dichloromethane are a slightly anodic and cathodic shift by 0.05 V and 0.06 V, respectively, compared to those in acetonitrile. Meanwhile, any obvious reduction wave did not appear within the potential window (−1.5 V–0 V versus Fc/Fc^+^). Thus, the HOMO energy levels (−[*E*_pa_^ox^ + 4.8] eV) versus vacuum level were estimated from the *E*_pa_^ox^, and the corresponding LUMO energy levels were estimated from the *E*_pa_^ox^ and the onsets (optical energy gap: *E*_g_^opt^) of the photoabsorption spectra in the acetonitrile or dichloromethane solution (Figure 5, Table 2). Both in acetonitrile and dichloromethane, the HOMO and LUMO energy levels (ca. −5.25 eV and ca. −2.80 eV, respectively) of **KK2** are higher than those (ca. −5.30 eV and ca. −3.15 eV, respectively) of **OD2**. It is worth noting here that the LUMO energy levels (−2.84 eV and −3.26 eV, respectively) of **KK2** and **OD2** in dichloromethane are much lower than those (−2.74 eV and −3.07 eV, respectively) in acetonitrile, although the HOMO energy levels (−5.27 eV and −5.26 eV, respectively) of **KK2** and **OD2** in dichloromethane are similar to those (−5.22 eV and −5.32 eV, respectively) in acetonitrile. Consequently, for both **KK2** and **OD2** the bathochromic shift of *λ*_max_^abs^ in halogenated solvents relative to those in non‐halogenated solvents is attributed to the stabilization of the LUMO energy level, leading to a decrease in the HOMO–LUMO band gap. In addition, it was revealed that the lowering in the LUMO energy level of **OD2** with a change in solvent from acetonitrile to dichloromethane is larger than that in **KK2**, resulting in the pronounced b‐OHC of **OD2**.

**Table 2:** Electrochemical data of **KK2** and **OD2** in acetonitrile and dichloromethane.

Dye	Solvent	$E_{\mathrm{pa}}^{\mathrm{ox}}$ ^a^ ^)^(V)	$E_{g}^{\mathrm{opt}}$ ^b^ ^)^(eV)	HOMO^c^ ^)^(eV)	LUMO (eV)
KK2	Acetonitrile	0.42	2.48	−5.22	−2.74
KK2	CH_2_Cl_2_	0.47	2.43	−5.27	−2.84
OD2	Acetonitrile	0.52	2.25	−5.32	−3.07
OD2	CH_2_Cl_2_	0.46	2.00	−5.26	−3.26

^a)^ Oxidation peak potential versus Fc/Fc^+^.

^b)^ Optical energy gaps ($E_{g}^{\mathrm{opt}}$) were determined from the onsets of the photoabsorption spectra in acetonitrile and dichloromethane.

^c)^ Versus vacuum level.

**Figure 6:**
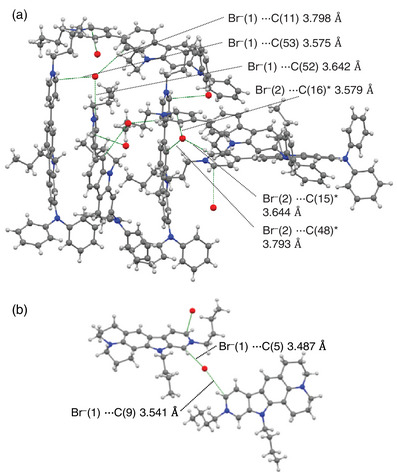
Crystal structures of (a) **OD2** and (b) **KK2**.

A single‐crystal X‐ray structural analysis was successfully made for **KK2** as well as **OD2** ^[^ ^57^ ^]^ (Figure 6, Table S7). There are two crystallographically independent dye molecules in the crystal structure of **OD2**. The bromide ion is located near pyridinium rings and its nearby carbazole part in a pair of dye molecules: the distances between Br^−^(1) and C(53) [or Br^−^(2) and C(16)*] in pyridinium ring and between Br^−^(1) and C(52) [or Br^−^(2) and C(15)*] in pyridinium ring are ca. 3.58 Å and 3.64 Å, respectively, and the distances between Br^−^(1) and C(11) in carbazole moiety and between Br^−^(2) and C(48) in carbazole moiety are ca. 3.80 Å and 3.79 Å, respectively. Similarly, for **KK2,** the bromide ion is located near pyridinium rings in two adjacent dye molecules: the distances between Br^−^(1) and C(5) in pyridinium ring and between Br^−^(1) and C(9) in pyridinium ring are ca. 3.49 Å and 3.54 Å, respectively.

**Figure 7:**
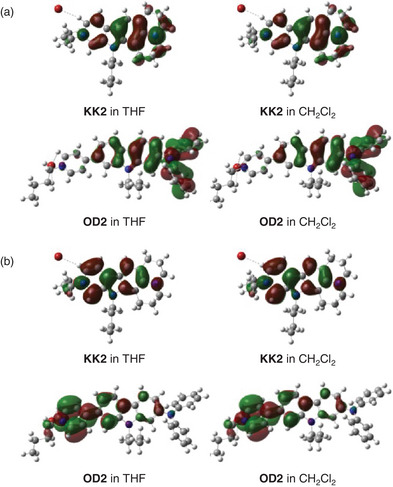
(a) HOMO and (b) LUMO of **KK2** and **OD2** derived from the DFT calculations by the self‐consistent reaction field (SCRF) method using the integral equation formalism polarizable continuum model (IEFPCM) based on B3LYP/6–31G+(d,p) level after geometrical optimizations at the M06−2X/6–31G+(d,p) level using the molecular structures of the single‐crystal X‐ray structural analysis.

Thus, in order to reveal the molecular orbitals of **KK2** and **OD2** in THF or dichloromethane, we performed the DFT calculations by the self‐consistent reaction field (SCRF) method using the integral equation formalism polarizable continuum model (IEFPCM) based on B3LYP/6–31G+(d,p) level after geometrical optimizations at M06−2X/6–31G+(d,p) level using the molecular structures derived from the single‐crystal X‐ray structural analysis.^[^ ^58^ ^]^ The DFT calculations demonstrated that for **KK2** in both THF and dichloromethane the HOMO and LUMO are delocalized over the whole molecule. In contrast, for **OD2** the HOMO is localized on the (diphenylamino)carbazole moiety and the LUMO is mostly localized on pyridinium rings and its nearby carbazole part, indicating that the HOMO and LUMO distributions for the molecular structure are adequately separated (Figure 7). Accordingly, the photoexcitation of **OD2** induces intense ICT characteristics from the (diphenylamino)carbazole unit as a D–π moiety to the pyridinium ring as an A moiety, compared to that of **KK2**. Furthermore, the DFT calculations at B3LYP/6–31G+(d,p) for the molecular structures with the XB (ClH_2_C─Cl···Br^−^) or the complex such as [ClH_2_C─Cl·Br]^−^ between dichloromethane and bromide ion which has been geometrically optimized at M06−2X/6–31G+(d,p) level, showed that the HOMO and LUMO distributions of **KK2** and **OD2** with the XB are similar to those without the XB (Figure S6). Indeed, for both **KK2** and **OD2**, one can see little change in the HOMO and LUMO energy levels between the dye molecule with and without the XB (Figure S7) that is not consistent with the experimental results from the CV and the photoabsorption spectral analyses (Figure 5). Thus, unfortunately, the DFT calculations in the current stage did not give a useful insight into the influence of XB on the expression of b‐OHC for D–A and D‐π‐A pyridinium dyes. Nevertheless, based on the experimental and theoretical results, it was suggested that the formation of XB or complex such as [R─X·Y]^−^ between the halogen atom of organohalogen molecule and the counter anion of the D–A or D‐π‐A pyridinium molecule induces the decrease in the ring current of pyridinium ring that the LUMO is mainly localized, resulting in expression of b‐OHC due to the stabilization of the LUMO energy level. Moreover, it was revealed that the pronounced b‐OHC of **OD2** relative to that of **KK2** is attributed to the well‐separated HOMO and LUMO distributions for **OD2**, leading to the intense ICT‐based photoabsorption and a great perturbation to the LUMO energy level by the formation of XB or [R─X·Y]^−^. Consequently, this work offers a deeper insight into the mechanism for the expression and the origin of OHC. Meanwhile, in order to fully clarify the b‐OHC with the objective of the XB, we would like to verify the formation of XB from the single‐crystal X‐ray structural analyses for the organohalogen molecules‐inclusion dye crystals, and then to perform the DFT calculation using the obtained molecular structure with the XB in the next work.

## 3 Conclusion

In this work, we attempted to elucidate the bathochromic shift‐type OHC (b‐OHC) of D–A and D‐π‐A pyridinium dyes bearing halide ion as a counter anion with the objective of the intermolecular interaction between the organohalogen and the dye molecules. It was revealed that there is a good relationship between the most positive surface electrostatic potential (*V*_S,max_) values associated with the most positive *σ*‐hole on halogen atoms in organohalogen molecule and the intramolecular charge transfer (ICT)‐based photoabsorption maximum wavenumbers (*v* ^∼^_max_^abs^), indicating that the formation of halogen bond (XB) or complex such as [R─X·Y]^−^ between the halogen atom (X) of organohalogen molecule and the counter anion (Y^−^) of dye molecule contributes to the expression of b‐OHC. The experimental and theoretical results revealed that the formation of XB or complex [R─X·Y]^−^ induces a decrease in the ring current of the pyridinium ring where the LUMO is mainly localized, resulting in the expression of b‐OHC due to the stabilization of the LUMO energy level. Moreover, the pronounced b‐OHC of D‐π‐A pyridinium dye relative to that of D–A pyridinium dye is attributed to the well‐separated HOMO and LUMO distributions for the D‐π‐A pyridinium dye, leading to the intense ICT‐based photoabsorption and a great perturbation to the LUMO energy level by the formation of XB or [R─X·Y]^−^. Consequently, we believe that this work contributes to a deeper insight into the mechanism for the expression and the origin of OHC, leading to the development of organic dye possessing hypsochromic shift‐type OHC (h‐OHC) as well as modulation of b‐OHC by selecting kinds of counter anions.

## 4 Experimental Section

### 4.1 General

Melting points were measured with an AS ONE ATM‐02. IR spectra were recorded using a SHIMADZU IRTracer‐100 spectrometer by the ATR method. ^1^H and ^13^C NMR spectra were recorded using a Varian‐500 FT NMR spectrometer. High‐resolution mass spectral data were obtained using ESI with a Thermo Fisher Scientific LTQ Orbitrap XL. Photoabsorption spectra were observed with a SHIMADZU UV‐3600 plus. Cyclic voltammetry (CV) curves were recorded in acetonitrile/Bu_4_NClO_4_ (0.1 M) or dichloromethane/Bu_4_NClO_4_ (0.1 M) solution with a three‐electrode system consisting of Ag/Ag^+^ as the reference electrode, a Pt plate as the working electrode, and a Pt wire as the counter electrode using an Electrochemical Measurement System HZ‐7000 (HOKUTO DENKO).

### 4.2 Theoretical Calculations

The Gaussian 16 program^[^ ^58^ ^]^ was used for density functional theory (DFT) calculations and second‐order Møller–Plesset theory (MP2). Geometry optimizations and frequency calculations of **KK2** and **OD2** were carried out at the M06−2X/6–31 + G(d,p) level with the integral equation formalism polarizable continuum model (IEFPCM) for THF or dichloromethane as a solvent using the molecular structures derived from the single‐crystal X‐ray structural analysis. No imaginary frequencies were found for any of the optimized structures. Single‐point energy calculations were subsequently performed at the B3LYP/6–31 + G(d,p) level with the integral equation formalism polarizable continuum model (IEFPCM) for THF or dichloromethane as a solvent. Geometry optimizations and frequency calculations of halogenated solvent molecules have been performed with the use of the MP2 method employing the aug‐cc‐pVDZ‐PP basis set for iodine and aug‐cc‐pVDZ‐PP for hydrogen, carbon, chlorine, and bromine atoms. The electrostatic potential maximum in this study was calculated by the Multiwfn software.^[^ ^59^, ^60^ ^]^

### 4.3 X‐ray Crystallographic Analysis

A single crystal of **KK2** was grown by slow diffusion of hexane into a solution of **KK2** in CH_2_Cl_2_ at room temperature as a yellow plate crystal, and was air stable. Diffraction measurement was made on a Rigaku XtaLAB Synergy‐R/DW HPC detector with Mo Kα radiation (λ = 0.71073 Å, 50 kV, 24 mA) at 100 K. Data reduction, integration, scaling, and space group determination were carried out using the CrysAlisPro (Rigaku Oxford Diffraction, 2021, 2023). The structural analysis was performed using WinGX.^[^ ^61^ ^]^ The crystal structure was solved by SHELXT‐2014/5 and refined by SHELXL‐2018/3.^[^ ^62^, ^63^ ^]^ All atoms were refined anisotropically. CCDC‐2448145 (**KK2**) contains the supplementary crystallographic data for this paper. Crystallographic data: C_25_H_34_BrN_3_, *M* = 456.46, monoclinic, *a* = 9.2397(2), *b* = 16.1343(4), *c* = 15.0485(4) Å, β = 93.457(2)°, *V* = 2239.29(9)Å^3^, *D*_calcd_ = 1.354 g cm^−3^, space group *P*2_1_/*c*, *Z* = 4, 16970 reflections measured, 5329 unique (*R*_int_ = 0.0256), which were used in all calculations. The final *R*_1_ (reflections) = 0.0341 (4425) [*I* > 2σ(*I*)], w*R*_2_(reflections) = 0.0871 (5329). GOF = 1.016.

Deposition Numbers 2448145 (**KK2**) and 928592 (**OD2**) contain the supplementary crystallographic data for this paper. These data are provided free of charge by the joint Cambridge Crystallographic Data Centre and Fachinformationszentrum Karlsruhe Access Structures service.

## Conflict of Interests

The authors declare no conflict of interest.

## Supporting information



Supporting Information

Supporting Information

## Data Availability

The data that support the findings of this study are available in the Supporting Information of this article.
