# Supplementary material for: Elucidation of Organohalogenochromism (OHC) of D–A Pyridinium and D‐π‐A Pyridinium Dyes: Effect of Halogen Bond
Source: Chem Asian J. 2025 Jun 23;20(18):e00746. doi: 10.1002/asia.202500746 (PMC12450044; doi:10.1002/asia.202500746)
Supplement: Supplementary file 1 — Supporting Information [file ASIA-20-e00746-s002.pdf]

Supporting Information

Elucidation of Organohalogenochromism (OHC) of D-A Pyridinium and D-π-A Pyridinium Dyes: Effect of Halogen Bond

Kumpei Kozuka,[a] Keiichi Imato[a] and Yousuke Ooyama*[a]

[a] Applied Chemistry Program, Graduate School of Advanced Science and Engineering, Hiroshima University, 1-4-1 Kagamiyama, Higashi-Hiroshima 739-8527, Japan

E-mail: yooyama@hiroshima-u.ac.jp

Synthesis

11,13-dibutyl-2,3,5,6,7,13-hexahydro-1*H*-pyrido[3,2,1-*ij*]pyrido[4',3':4,5]pyrrolo[2,3-*f*]quinolin-11-ium bromide (KK2)

A solution of **ET-1**[1] (0.101 g, 0.316 mmol) and 1-bromobutane (2.62 mL, 25.0 mmol) in dry acetonitrile (25 mL) was stirred for 20 h at 80°C. After concentrating under reduced pressure, the resulting residue was subjected to reprecipitation from MeOH/diethyl ether to give **KK2** (0.126 g, 87% yield) as orange solids; m.p. 230–232 °C ; FT-IR (ATR): $\tilde{\nu}$ = 1610, 1302 cm^{-1}, ^{1}H NMR (500 MHz, $CDCl_3$, ppm): δ = 0.92–0.99 (m, 6H, CH_3), 1.43–1.53 (m, 4H, CH_2), 1.79–1.85 (m, 2H, CH_2), 1.98–2.11 (m, 6H, CH_2), 2.90 (t, J = 6.27 Hz, 2H, CH_2), 3.20 (t, J = 6.39 Hz, 2H, CH_2), 3.36–3.39 (m, 4H, CH_2), 4.80 (t, J = 7.68 Hz, 2H, CH_2), 4.87 (t, J = 7.37, 2H, CH_2), 7.51 (s, 1H, aromatic), 7.79 (d, J = 6.41 Hz, 1H, aromatic), 7.88 (d, J = 6.55, 1H, aromatic), 10.36 (s, 1H, aromatic); ^{13}C NMR (125 MHz, $CDCl_3$, ppm): δ = 13.73, 14.11, 19.50, 20.06, 21.18, 21.61, 23.34, 28.85, 33.21, 34.24, 46.83, 49.81, 51.25, 60.08, 101.47, 110.27, 112.82, 120.30, 120.48, 126.20, 131.18, 132.57, 137.14, 144.30, 147.62; HRMS (ESI): *m/z* found 376.27478 $[M–Br]^+$, calculated for $C_{25}H_{34}N_3$ $[M–Br]^+$: 376.27472.

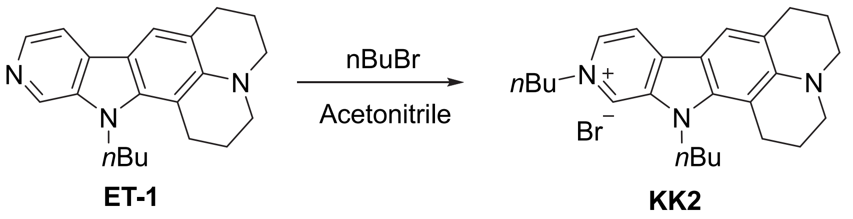

Scheme S1 Synthesis of **KK2**.

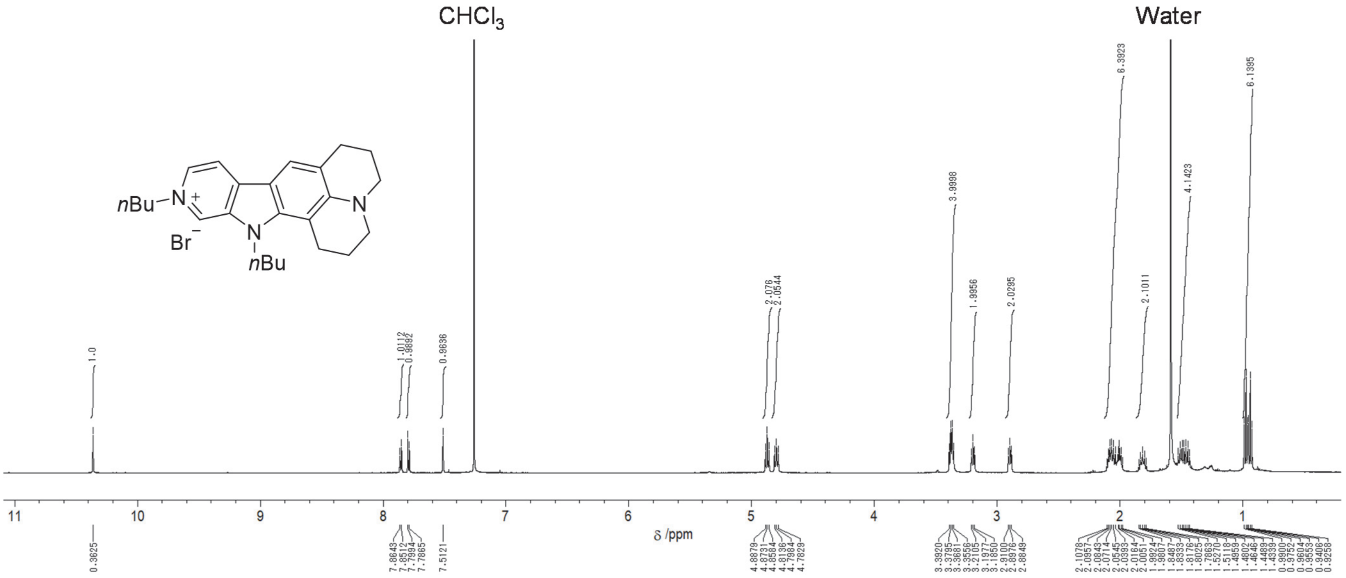

Figure S1 ^{1}H NMR (500MHz) spectrum of **KK2** in $CDCl_3$.

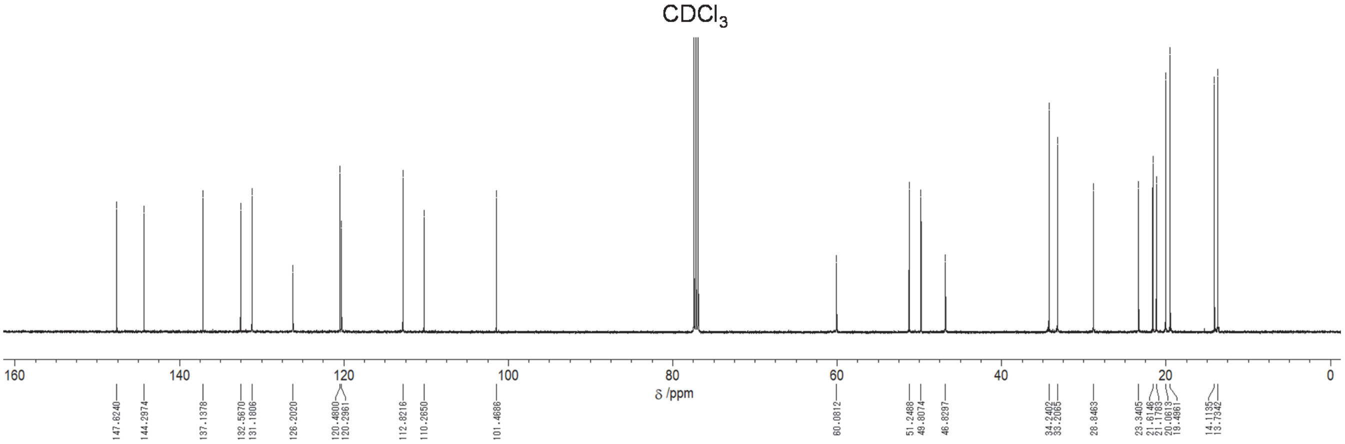

Figure S2 ^{13}C NMR (125MHz) spectrum of **KK2** in $CDCl_3$.

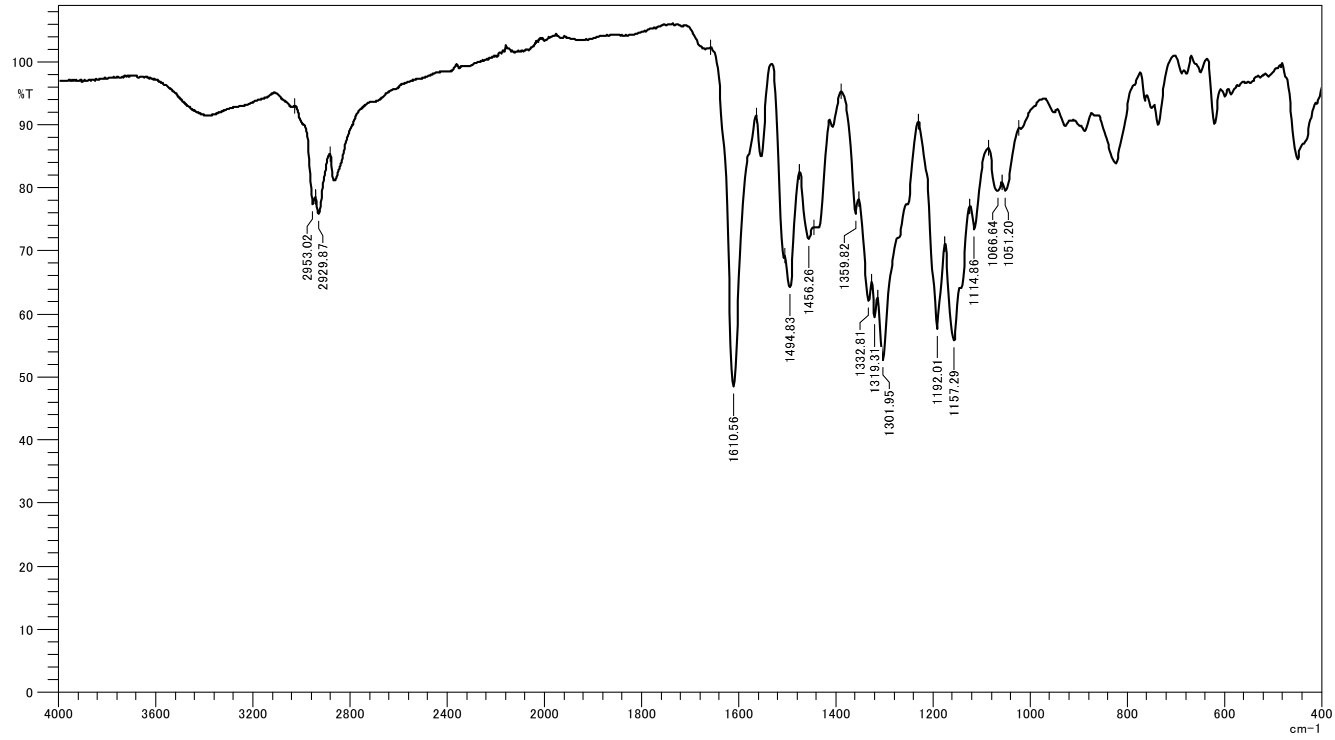

Figure S3 FT-IR (ATR) spectrum of **KK2**.

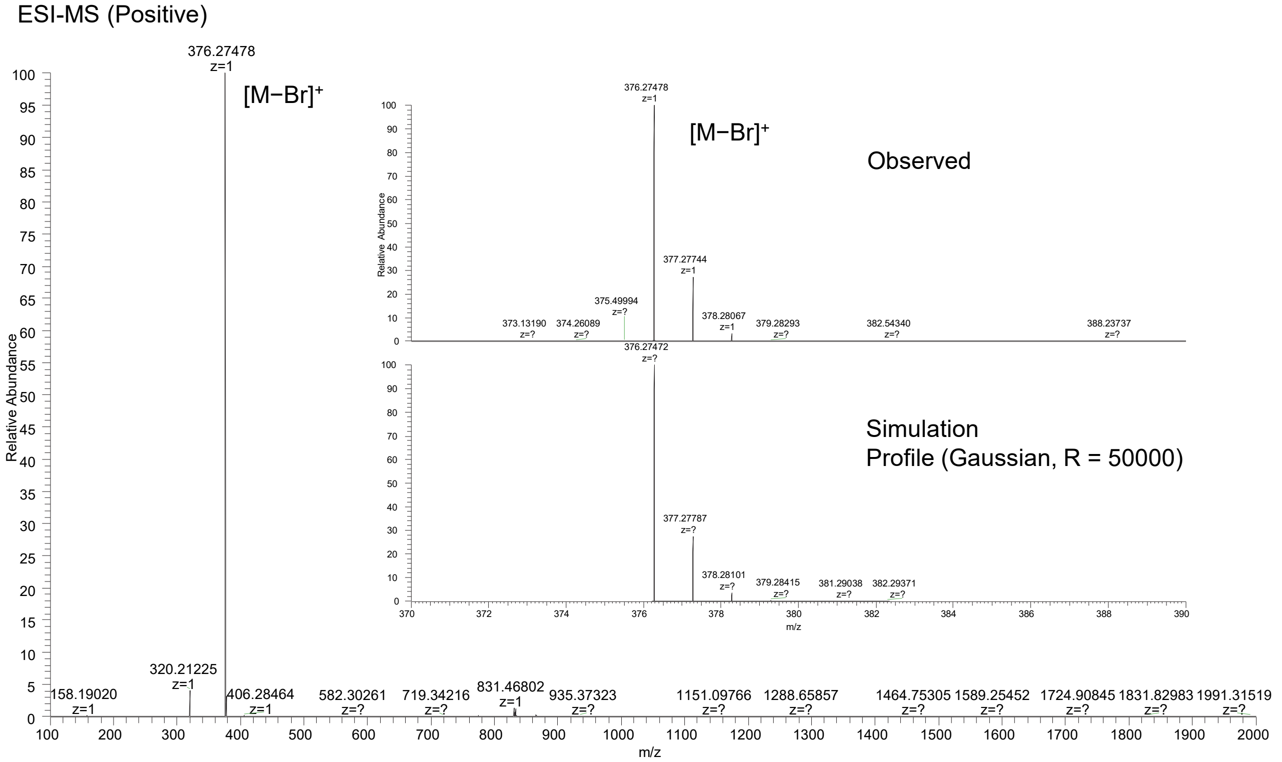

Figure S4 HRMS spectrum of **KK2**.

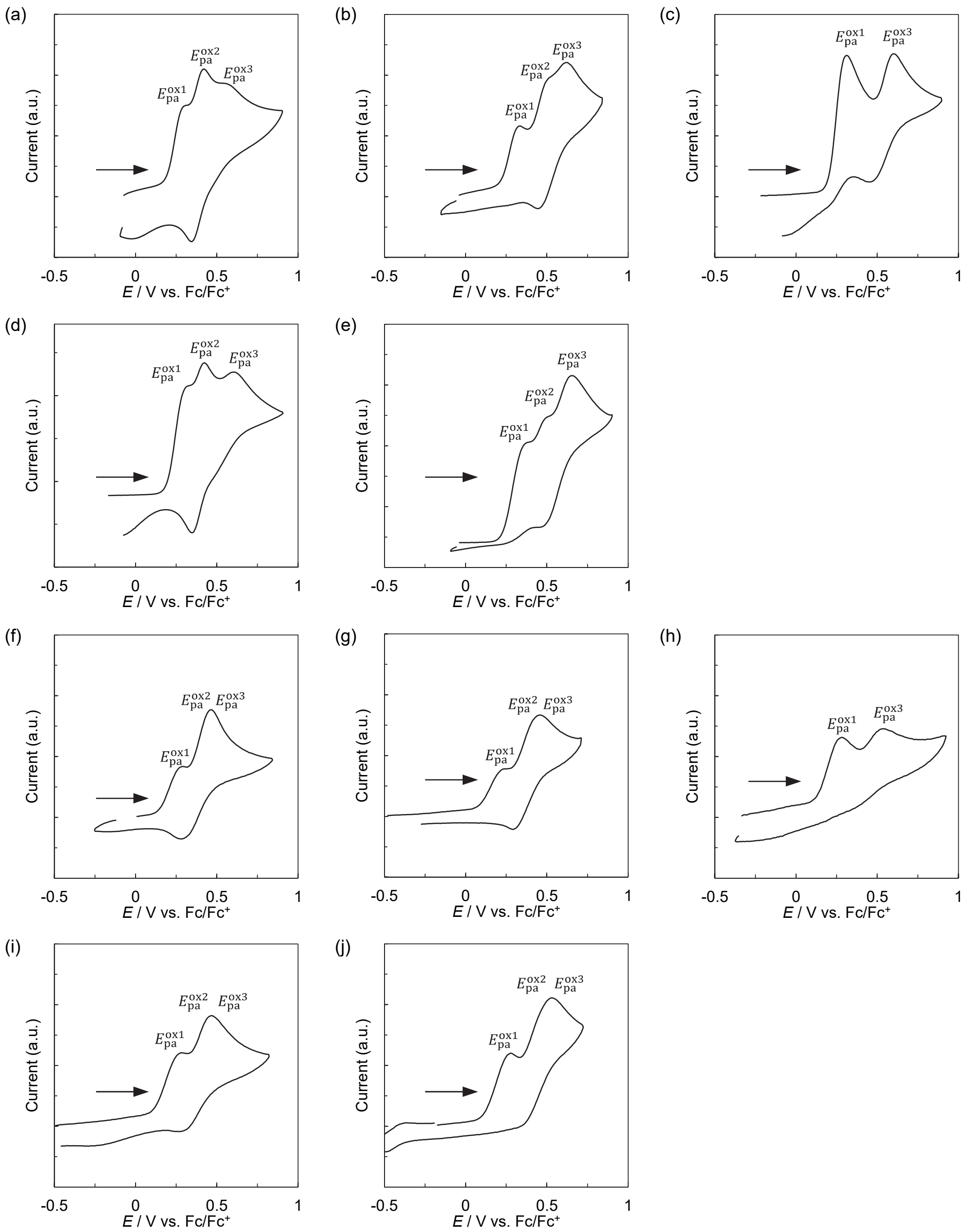

Figure S5 Cyclic voltammograms of (a) **KK2** (1 mM), (b) **OD2** (1 mM), (c) tetrabutylammonium bromide (Bu_4NBr, 1 mM), (d) **KK2** (1 mM) and Bu_4NBr (1 mM), and (e) **OD2** (1 mM) and Bu_4NBr (1 mM) in acetonitrile containing 0.1 M tetrabutylammonium perchlorate (Bu_4NClO_4), and (f) **KK2** (1 mM), (g) **OD2** (1 mM) (h) Bu_4NBr (1 mM), (i) **KK2** (1 mM) and Bu_4NBr (1 mM), and (j) **OD2** (1 mM) and Bu_4NBr (1 mM) in dichloromethane containing 0.1 M Bu_4NClO_4 at a scan rate of 100 mV s^{-1}. The E_{pa}^{ox2} is attributed to the dye, while the E_{pa}^{ox1} and E_{pa}^{ox3} correspond to the oxidation of Br^{-}[2]. The arrow denotes the direction of the potential scan.

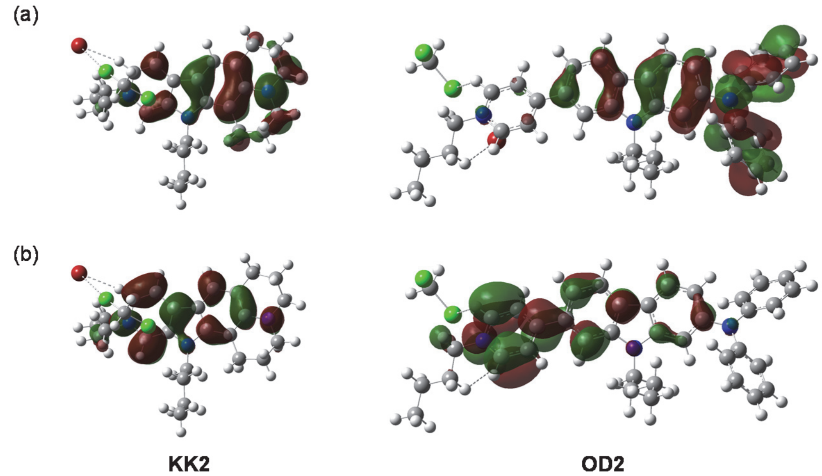

Figure S6 (a) HOMO and (b) LUMO of **KK2** and **OD2** with the halogen bond (XB: ClH2C–Cl···Br⁻) or the complex [ClH2C–Cl·Br]⁻ between bromide ion and dichloromethane, derived from the DFT calculations by the self-consistent reaction field (SCRF) method using the integral equation formalism polarizable continuum model (IEFPCM) based on B3LYP/6-31G+(d,p) level after geometrical optimizations at the M062X/6-31G+(d,p) level.

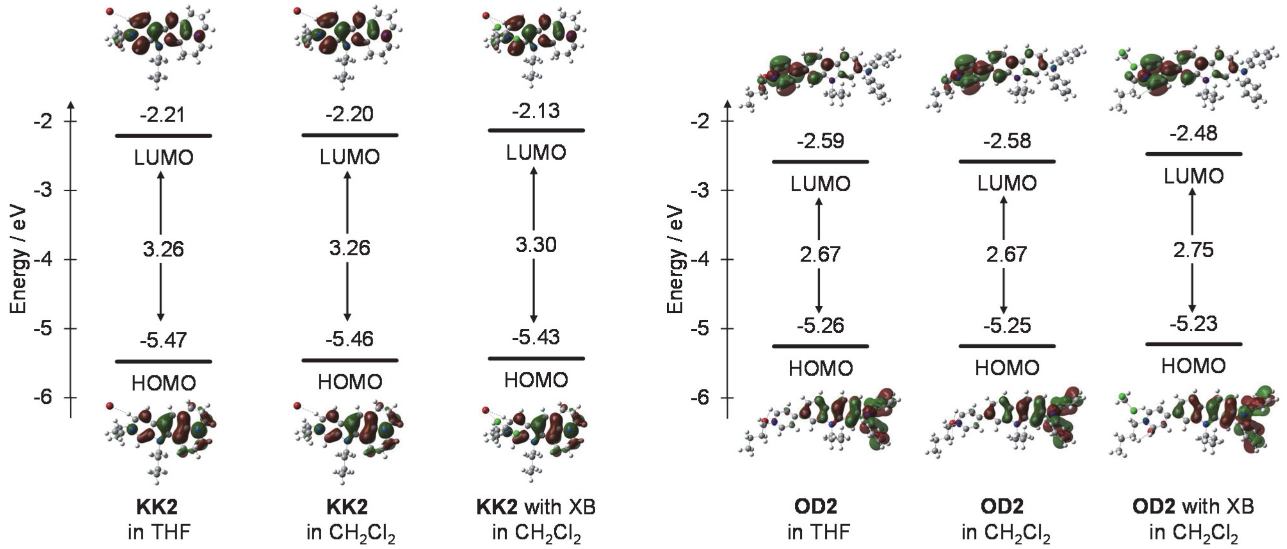

Figure S7 Energy level diagram, HOMO and LUMO of **KK2** and **OD2** in THF and dichloromethane and **KK2** and **OD2** with the halogen bond (XB: ClH2C–Cl···Br⁻) or the complex [ClH2C–Cl·Br]⁻in dichloromethane derived from DFT calculations at the B3LYP/6-31+G(d,p) level with the IEFPCM solvation model.

Table S1 Geometrical coordinates of the optimized **KK2** in THF by DFT at the M062X/6-31+G(d,p) level.

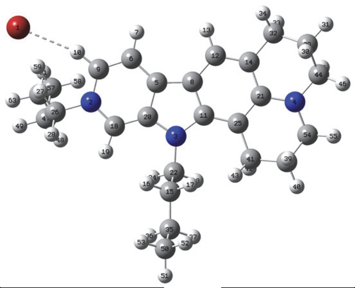

Cartesian coordinates

Tag	Symbol	X	Y	Z
1	Br	-6.1182014	-1.6965355	-0.9982953
2	N	-2.6798428	0.5524386	-0.2815566
3	N	0.9116602	1.1301189	0.209851
4	N	5.262087	-1.2410976	0.2684468
5	C	-0.2740265	-0.7812574	-0.2186362
6	C	-1.457318	-1.4854023	-0.4520419
7	H	-1.4687721	-2.5562672	-0.615919
8	C	1.1053516	-1.1199554	-0.1246059
9	C	-2.6491658	-0.7890201	-0.4762233
10	H	-3.6124109	-1.2604537	-0.6555218
11	C	1.828246	0.0745996	0.1247285
12	C	1.7859698	-2.3404311	-0.2247595
13	H	1.2348651	-3.2584633	-0.4135725
14	C	3.1498755	-2.3885308	-0.0693883
15	C	1.2571395	3.381095	-0.8021644
16	H	0.3306355	3.3002605	-1.3852936
17	H	2.053875	2.9630929	-1.4305865
18	C	-1.5624007	1.2769866	-0.0479125
19	H	-1.6968934	2.3435102	0.0887482
20	C	-0.3474713	0.6258179	-0.0114558
21	C	3.8872366	-1.1723221	0.161556
22	C	1.1284513	2.5491371	0.4728721
23	H	2.0065783	2.6578301	1.1075549
24	H	0.2808431	2.90162	1.070967
25	C	3.2270546	0.0822545	0.247494
26	C	-3.9904367	1.2478597	-0.2442527
27	H	-4.7085588	0.5920912	-0.7414599
28	H	-3.8831845	2.1709052	-0.8181825
29	C	5.3182921	-3.5641567	-0.5135368
30	H	5.4051035	-3.2311332	-1.5539275
31	H	5.8551282	-4.511442	-0.4157984
32	C	3.8559186	-3.7258854	-0.1262217
33	H	3.8015644	-4.2070806	0.8592087
34	H	3.3309742	-4.3817916	-0.826918
35	C	1.5517576	4.8473769	-0.4924907
36	H	0.7618946	5.2478538	0.155467
37	H	2.48673	4.9129775	0.0788865
38	C	5.4922205	1.1727907	0.097302
39	H	5.6188562	1.0791988	-0.9867533
40	H	6.0723047	2.0369208	0.4313013
41	C	4.0207238	1.361037	0.4432261
42	H	3.9421198	1.6989712	1.4846091
43	H	3.6109295	2.1571437	-0.1834186
44	C	5.9438756	-2.5237209	0.397685
45	H	5.9160758	-2.8732572	1.4431991
46	H	6.9924593	-2.3605728	0.1344617
47	C	-4.4248987	1.5233111	1.1936228
48	H	-3.7204445	2.2168909	1.6728146
49	H	-5.3891075	2.0427128	1.1378129
50	C	1.6601102	5.6955198	-1.757771
51	H	1.8755308	6.7401543	-1.5187487
52	H	2.4603487	5.325569	-2.4067054
53	H	0.7260664	5.6652198	-2.3277857
54	C	6.0034965	-0.0909015	0.7609679
55	H	7.0589837	-0.252498	0.5280813
56	H	5.9127551	-0.0101014	1.8571449
57	C	-4.5719681	0.2534901	2.0313323
58	H	-3.597815	-0.2460616	2.1155445
59	H	-5.2382147	-0.4408701	1.5058173
60	C	-5.1058855	0.5483585	3.430985
61	H	-5.1938046	-0.3682619	4.0200902
62	H	-4.4422136	1.2335017	3.9695542
63	H	-6.0963764	1.0120489	3.3806121

Table S2 Geometrical coordinates of the optimized **KK2** in dichloromethane by DFT at the M062X/6-31+G(d,p) level.

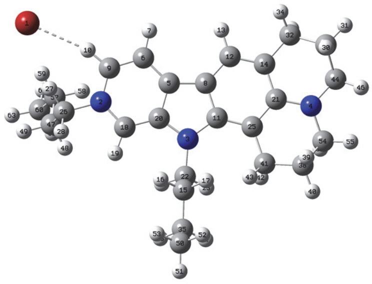

Cartesian coordinates

Tag	Symbol	X	Y	Z
1	Br	-6.1182014	-1.6965355	-0.9982953
2	N	-2.6798428	0.5524386	-0.2815566
3	N	0.9116602	1.1301189	0.209851
4	N	5.262087	-1.2410976	0.2684468
5	C	-0.2740265	-0.7812574	-0.2186362
6	C	-1.457318	-1.4854023	-0.4520419
7	H	-1.4687721	-2.5562672	-0.615919
8	C	1.1053516	-1.1199554	-0.1246059
9	C	-2.6491658	-0.7890201	-0.4762233
10	H	-3.6124109	-1.2604537	-0.6555218
11	C	1.828246	0.0745996	0.1247285
12	C	1.7859698	-2.3404311	-0.2247595
13	H	1.2348651	-3.2584633	-0.4135725
14	C	3.1498755	-2.3885308	-0.0693883
15	C	1.2571395	3.381095	-0.8021644
16	H	0.3306355	3.3002605	-1.3852936
17	H	2.053875	2.9630929	-1.4305865
18	C	-1.5624007	1.2769866	-0.0479125
19	H	-1.6968934	2.3435102	0.0887482
20	C	-0.3474713	0.6258179	-0.0114558
21	C	3.8872366	-1.1723221	0.161556
22	C	1.1284513	2.5491371	0.4728721
23	H	2.0065783	2.6578301	1.1075549
24	H	0.2808431	2.90162	1.070967
25	C	3.2270546	0.0822545	0.247494
26	C	-3.9904367	1.2478597	-0.2442527
27	H	-4.7085588	0.5920912	-0.7414599
28	H	-3.8831845	2.1709052	-0.8181825
29	C	5.3182921	-3.5641567	-0.5135368
30	H	5.4051035	-3.2311332	-1.5539275
31	H	5.8551282	-4.511442	-0.4157984
32	C	3.8559186	-3.7258854	-0.1262217
33	H	3.8015644	-4.2070806	0.8592087
34	H	3.3309742	-4.3817916	-0.826918
35	C	1.5517576	4.8473769	-0.4924907
36	H	0.7618946	5.2478538	0.155467
37	H	2.48673	4.9129775	0.0788865
38	C	5.4922205	1.1727907	0.097302
39	H	5.6188562	1.0791988	-0.9867533
40	H	6.0723047	2.0369208	0.4313013
41	C	4.0207238	1.361037	0.4432261
42	H	3.9421198	1.6989712	1.4846091
43	H	3.6109295	2.1571437	-0.1834186
44	C	5.9438756	-2.5237209	0.397685
45	H	5.9160758	-2.8732572	1.4431991
46	H	6.9924593	-2.3605728	0.1344617
47	C	-4.4248987	1.5233111	1.1936228
48	H	-3.7204445	2.2168909	1.6728146
49	H	-5.3891075	2.0427128	1.1378129
50	C	1.6601102	5.6955198	-1.757771
51	H	1.8755308	6.7401543	-1.5187487
52	H	2.4603487	5.325569	-2.4067054
53	H	0.7260664	5.6652198	-2.3277857
54	C	6.0034965	-0.0909015	0.7609679
55	H	7.0589837	-0.252498	0.5280813
56	H	5.9127551	-0.0101014	1.8571449
57	C	-4.5719681	0.2534901	2.0313323
58	H	-3.597815	-0.2460616	2.1155445
59	H	-5.2382147	-0.4408701	1.5058173
60	C	-5.1058855	0.5483585	3.430985
61	H	-5.1938046	-0.3682619	4.0200902
62	H	-4.4422136	1.2335017	3.9695542
63	H	-6.0963764	1.0120489	3.3806121

Table S3 Geometrical coordinates of the optimized **KK2** with the halogen bond (XB: $ClH_2C–Cl\cdots Br^{-}$) or the complex $[ClH_2C–Cl\cdot Br]^{-}$ in dichloromethane by DFT at the M062X/6-31+G(d,p) level.

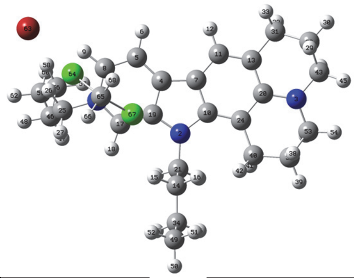

Cartesian coordinates

Tag	Symbol	X	Y	Z
1	N	-2.282329	0.3564646	0.6054277
2	N	1.3303624	0.9546564	0.8387568
3	N	5.6961975	-1.2571167	0.0525959
4	C	0.1333488	-0.9063923	0.2511034
5	C	-1.0560276	-1.5963005	0.0130023
6	H	-1.0681742	-2.6277301	-0.3187383
7	C	1.5202376	-1.2148412	0.1561128
8	C	-2.2544863	-0.9334668	0.1914028
9	H	-3.2241182	-1.3923679	0.0189693
10	C	2.2478893	-0.0458948	0.4953343
11	C	2.202548	-2.3869542	-0.1919669
12	H	1.64691	-3.2857681	-0.4481459
13	C	3.5760402	-2.4121653	-0.2044297
14	C	1.4982934	3.3090783	0.0554145
15	H	0.500684	3.2700918	-0.4020562
16	H	2.2049257	2.9766916	-0.7161232
17	C	-1.1592062	1.0632937	0.8576288
18	H	-1.2936982	2.0919968	1.1716001
19	C	0.0620953	0.4493856	0.6766008
20	C	4.3160544	-1.2182491	0.1144958
21	C	1.5487628	2.3412016	1.2366667
22	H	2.4964903	2.4101296	1.768015
23	H	0.7747602	2.5957268	1.9691449
24	C	3.6517606	-0.0105581	0.4558264
25	C	-3.59089	0.9943492	0.8804707
26	H	-4.340437	0.4234796	0.3291343
27	H	-3.5489425	2.0101881	0.4798507
28	C	5.6986835	-3.4484902	-1.0455918
29	H	5.6666293	-2.9720929	-2.0319353
30	H	6.2566671	-4.3838528	-1.1400107
31	C	4.2899649	-3.7060509	-0.5318704
32	H	4.3505778	-4.3254228	0.3727195
33	H	3.6998744	-4.2695112	-1.2606851
34	C	1.8212329	4.7404212	0.4782893
35	H	1.1289332	5.0498168	1.2713589
36	H	2.8279587	4.7675535	0.9149043
37	C	5.869436	1.1610364	0.2149209
38	H	5.8779962	1.2261587	-0.8785489
39	H	6.4698627	1.9862049	0.6065814
40	C	4.4416596	1.2545248	0.7382393
41	H	4.4710316	1.4483749	1.8182804
42	H	3.9531696	2.1139262	0.2716253
43	C	6.4048043	-2.5256529	-0.0688963
44	H	6.4938828	-3.0157969	0.915075
45	H	7.4168522	-2.2988292	-0.4149631
46	C	-3.8940817	0.9816367	2.3778584
47	H	-3.2159964	1.6671171	2.9032797
48	H	-4.90689	1.3841075	2.4990537
49	C	1.7391897	5.7201582	-0.6902497
50	H	1.9784938	6.7385299	-0.3732431
51	H	2.4403404	5.4393038	-1.4826813
52	H	0.7329748	5.7280571	-1.1213718
53	C	6.4685503	-0.1684748	0.6297213
54	H	7.4955008	-0.2622337	0.2676473
55	H	6.4939803	-0.2494212	1.7295468
56	C	-3.8131337	-0.4112275	3.0050494
57	H	-2.779326	-0.7767809	2.9545848
58	H	-4.422806	-1.1073355	2.4157161
59	C	-4.274338	-0.404216	4.4607387
60	H	-4.1930507	-1.3989914	4.9065964
61	H	-3.6692591	0.2845314	5.0601578
62	H	-5.3188234	-0.0855255	4.5367853
63	Br	-5.6935416	-1.7845803	-0.3149861
64	Cl	-3.7628074	0.2432237	-2.575517
65	C	-2.5279596	1.1483616	-3.4735056
66	H	-2.9214933	2.1307213	-3.7163432
67	Cl	-1.0412768	1.3778803	-2.5103241
68	H	-2.2642837	0.5853881	-4.3636274

Table S4 Geometrical coordinates of the optimized **OD2** in THF by DFT at the M062X/6-31+G(d,p) level.

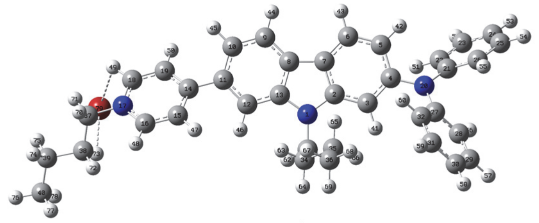

Cartesian coordinates

Tag	Symbol	X	Y	Z
1	N	1.0149918	0.4585313	0.3092531
2	C	2.1846906	-0.1796768	-0.060086
3	C	3.4908162	0.0901674	0.3522431
4	C	4.5186329	-0.6754219	-0.1958852
5	C	4.2348626	-1.7156691	-1.1136917
6	C	2.9326477	-1.9910774	-1.4931868
7	C	1.8864629	-1.2157627	-0.9764315
8	C	0.4574796	-1.1874086	-1.1749177
9	C	-0.4362002	-1.9410772	-1.9412679
10	C	-1.7892595	-1.6382256	-1.894326
11	C	-2.2702303	-0.5739846	-1.1009797
12	C	-1.3906042	0.1945476	-0.3312719
13	C	-0.0357785	-0.1273855	-0.370075
14	C	-3.711935	-0.2643685	-1.0855182
15	C	-4.1728159	1.0573161	-0.9425748
16	C	-5.5224723	1.3171757	-0.9187804
17	N	-6.427528	0.3240749	-1.0488006
18	C	-6.022609	-0.9516893	-1.1943446
19	C	-4.6780473	-1.2677973	-1.2241317
20	N	5.8609525	-0.3934865	0.1458984
21	C	6.7795536	-1.4528788	0.3532103
22	C	6.3807525	-2.6180363	1.0192196
23	C	7.2868033	-3.6582743	1.2114071
24	C	8.6019206	-3.5447598	0.7602252
25	C	9.0014868	-2.3791534	0.1047325
26	C	8.0976024	-1.3418051	-0.1069571
27	C	6.2716177	0.9473992	0.3557818
28	C	7.1270036	1.2704173	1.4162562
29	C	7.5327059	2.5886222	1.6123654
30	C	7.0798366	3.6030548	0.768972
31	C	6.2208082	3.2824047	-0.2834338
32	C	5.8244971	1.9654517	-0.4974743
33	C	0.905007	1.6243671	1.1720632
34	C	0.8832481	2.9413997	0.3943679
35	C	2.1658397	3.206129	-0.3937904
36	C	2.124076	4.5431524	-1.1305065
37	C	-7.8760835	0.6442909	-0.9839565
38	C	-8.2458951	1.3538866	0.313455
39	C	-9.7635479	1.416794	0.4770303
40	C	-10.1583352	2.0894575	1.7895781
41	H	3.7177757	0.8865017	1.0525715
42	H	5.0573081	-2.2899935	-1.5264248
43	H	2.7315723	-2.7867572	-2.2043646
44	H	-0.0792581	-2.7514899	-2.5691403
45	H	-2.4826943	-2.2052537	-2.5064644
46	H	-1.7593183	0.9961796	0.3009921
47	H	-3.4825779	1.889055	-0.8693323
48	H	-5.9197419	2.3197875	-0.8215436
49	H	-6.7984698	-1.7003333	-1.2707464
50	H	-4.3982513	-2.310409	-1.3126084
51	H	5.3606412	-2.7036215	1.3811999
52	H	6.9636776	-4.5565753	1.7283158
53	H	9.3071274	-4.3541336	0.9175323
54	H	10.0203825	-2.2793823	-0.256449
55	H	8.4067849	-0.4397357	-0.6263105
56	H	7.4730608	0.4854586	2.0816594
57	H	8.1972609	2.8226846	2.4382823
58	H	7.3922048	4.6297254	0.9286133
59	H	5.8643052	4.0601637	-0.9520958
60	H	5.1607029	1.7194704	-1.3212296
61	H	1.7485701	1.6038323	1.8684083
62	H	-0.0029974	1.5128097	1.7727926
63	H	0.0244516	2.9411256	-0.290299
64	H	0.7160428	3.7540135	1.1123717
65	H	2.3279568	2.3940037	-1.1137627
66	H	3.0243498	3.1890453	0.2915034
67	H	1.2883367	4.5719905	-1.8374544
68	H	3.0455622	4.7161656	-1.6933438
69	H	1.9970508	5.3730968	-0.4277244
70	H	-8.1168829	1.2477878	-1.8640124
71	H	-8.4019753	-0.3090272	-1.0474619
72	H	-7.8361152	2.3713362	0.3271457
73	H	-7.8120881	0.7937442	1.1476644
74	H	-10.2084158	1.9542161	-0.3700336
75	H	-10.1603869	0.3945293	0.4535203
76	H	-11.2444443	2.1206282	1.9091519
77	H	-9.7836197	3.1174816	1.8320644
78	H	-9.739204	1.5418111	2.639706
79	Br	-7.7207093	-1.9975217	1.3922552

Table S5 Geometrical coordinates of the optimized **OD2** in dichloromethane by DFT at the M062X/6-31+G(d,p) level.

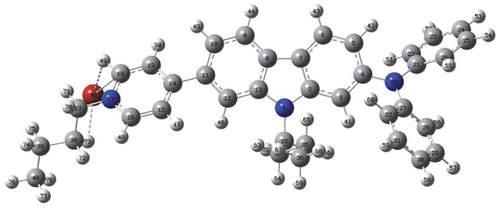

Cartesian coordinates

Tag	Symbol	X	Y	Z
1	N	1.0155183	0.4587892	0.3103
2	C	2.1850142	-0.1794128	-0.0593893
3	C	3.4912247	0.0896771	0.3533827
4	C	4.5189056	-0.6755751	-0.1954528
5	C	4.2349989	-1.7148856	-1.1143304
6	C	2.9327395	-1.9897244	-1.4942047
7	C	1.8866787	-1.2146523	-0.9767618
8	C	0.4577318	-1.1856535	-1.1755369
9	C	-0.4360524	-1.9381931	-1.9429954
10	C	-1.7889877	-1.6347273	-1.8961581
11	C	-2.2697259	-0.5710485	-1.1017953
12	C	-1.3900117	0.1963432	-0.3310505
13	C	-0.0352836	-0.1261748	-0.3698305
14	C	-3.7113354	-0.2607316	-1.0864772
15	C	-4.1716597	1.0606802	-0.9405209
16	C	-5.521267	1.3212995	-0.9171694
17	N	-6.426664	0.3288654	-1.0499193
18	C	-6.0221801	-0.9466707	-1.1987759
19	C	-4.6779157	-1.2634981	-1.2282467
20	N	5.8613437	-0.3941842	0.1465487
21	C	6.7798957	-1.4539314	0.3522034
22	C	6.3813293	-2.6197433	1.0172663
23	C	7.2873133	-3.66039	1.2077876
24	C	8.602187	-3.5466744	0.755824
25	C	9.0015542	-2.3804291	0.101269
26	C	8.0976993	-1.3426603	-0.108702
27	C	6.2723815	0.9464278	0.3576693
28	C	7.1284787	1.2680411	1.4180277
29	C	7.5346493	2.5859348	1.6154323
30	C	7.0814947	3.6014724	0.7734597
31	C	6.2216969	3.282239	-0.278786
32	C	5.8249496	1.9655926	-0.4941148
33	C	0.9058174	1.6248424	1.1730907
34	C	0.8844453	2.9417293	0.3951867
35	C	2.1669581	3.2057593	-0.3933193
36	C	2.1259538	4.5430347	-1.1296346
37	C	-7.8750702	0.649817	-0.9863234
38	C	-8.2464518	1.3541723	0.3134888
39	C	-9.7643021	1.4188823	0.4746357
40	C	-10.160338	2.0851927	1.7900595

Tag	Symbol	X	Y	Z
41	H	3.7181753	0.8853411	1.0544761
42	H	5.057297	-2.2889645	-1.52771
43	H	2.7315582	-2.7846354	-2.2062083
44	H	-0.0792925	-2.748014	-2.5717384
45	H	-2.4823799	-2.200741	-2.5092602
46	H	-1.7583543	0.9978691	0.3015331
47	H	-3.4812861	1.8920513	-0.8647745
48	H	-5.9178387	2.324012	-0.8182102
49	H	-6.7983538	-1.6946329	-1.2786926
50	H	-4.3986242	-2.305936	-1.3203735
51	H	5.361462	-2.705564	1.3798863
52	H	6.9643535	-4.5591644	1.7239781
53	H	9.3073368	-4.3563553	0.9118041
54	H	10.0202223	-2.280468	-0.2604989
55	H	8.406806	-0.4401489	-0.6273373
56	H	7.4747217	0.4823259	2.0824446
57	H	8.1997092	2.8188757	2.44126
58	H	7.3941296	4.6278977	0.9341536
59	H	5.8647597	4.0608516	-0.9462172
60	H	5.1604181	1.7209276	-1.3176677
61	H	1.7493341	1.6041017	1.8694544
62	H	-0.0022851	1.5136667	1.7736809
63	H	0.0253741	2.9419173	-0.289102
64	H	0.7179204	3.7543774	1.11327
65	H	2.3283185	2.393783	-1.1136555
66	H	3.0256549	3.187845	0.2917214
67	H	1.290008	4.5726374	-1.8363084
68	H	3.0474648	4.7155547	-1.6926039
69	H	1.9996179	5.3727818	-0.4264921
70	H	-8.1137659	1.2574595	-1.8640121
71	H	-8.4017183	-0.3026961	-1.0558493
72	H	-7.835212	2.3708617	0.332186
73	H	-7.8147099	0.7903651	1.1463159
74	H	-10.2064523	1.9617564	-0.3702758
75	H	-10.1633623	0.3975937	0.4444939
76	H	-11.2465985	2.1174971	1.9079722
77	H	-9.784016	3.1123317	1.8384657
78	H	-9.7433992	1.5326613	2.6381743
79	Br	-7.7248422	-2.0032138	1.3939419

Table S6 Geometrical coordinates of the optimized **OD2** with the halogen bond (XB: $ClH_2C–Cl\cdots Br^-$) or the complex $[ClH_2C–Cl\cdot Br]^-$ in dichloromethane by DFT at the M062X/6-31+G(d,p) level.

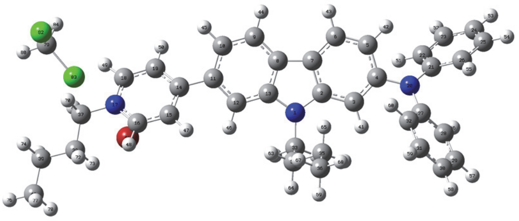

Cartesian coordinates

Tag	Symbol	X	Y	Z
1	N	1.9353126	0.5530217	0.2216057
2	C	3.0729886	-0.1620629	-0.1026417
3	C	4.3932905	0.0801782	0.2812438
4	C	5.3813324	-0.771066	-0.2100589
5	C	5.0456271	-1.8650347	-1.0430286
6	C	3.7291687	-2.1108476	-1.3941431
7	C	2.7226491	-1.2519346	-0.9348252
8	C	1.2942818	-1.1754548	-1.1289907
9	C	0.3623431	-1.9463603	-1.8303054
10	C	-0.9756224	-1.5797821	-1.8027479
11	C	-1.4014784	-0.4375608	-1.0901813
12	C	-0.4847674	0.3464618	-0.3835213
13	C	0.8548113	-0.0363846	-0.4060416
14	C	-2.8273598	-0.0599788	-1.0895411
15	C	-3.228343	1.283109	-1.0804022
16	C	-4.5675741	1.6097044	-1.0376987
17	N	-5.5117782	0.6533357	-1.0149845
18	C	-5.1587205	-0.6461149	-1.031209
19	C	-3.8382085	-1.0304796	-1.0829508
20	N	6.7382244	-0.5228466	0.1033989
21	C	7.6047583	-1.6014584	0.4088029
22	C	7.1497311	-2.6824494	1.1737934
23	C	8.0040126	-3.7441891	1.4617588
24	C	9.3231747	-3.7356149	1.0081604
25	C	9.7789608	-2.6536681	0.2532769
26	C	8.926877	-1.5965797	-0.0538836
27	C	7.2122022	0.8108269	0.1820268
28	C	8.0924844	1.1937462	1.2014879
29	C	8.5589829	2.5046982	1.2685223
30	C	8.1433271	3.4544117	0.3352824
31	C	7.2592104	3.0749535	-0.6760266
32	C	6.8017624	1.7631789	-0.7610367
33	C	1.8807175	1.7855322	0.9925495
34	C	1.8962047	3.0369427	0.1132469
35	C	3.1755339	3.1869954	-0.7095381
36	C	3.1743657	4.4611476	-1.5513308
37	C	-6.9550622	0.9724778	-0.8462145
38	C	-7.2301951	2.3344472	-0.228548
39	C	-8.6899921	2.4117839	0.2227345
40	C	-9.0333192	3.7810834	0.803713
41	H	4.6616354	0.9171741	0.916601
42	H	5.8391776	-2.5055096	-1.4129986
43	H	3.487558	-2.9493553	-2.0405795
44	H	0.678771	-2.8183945	-2.3942222
45	H	-1.700396	-2.1600637	-2.3645431
46	H	-0.8162966	1.2064774	0.1901035
47	H	-2.501983	2.0863622	-1.1113168
48	H	-4.9069747	2.6359383	-1.0147889
49	H	-5.9708325	-1.3600309	-0.9647509
50	H	-3.6045745	-2.0878864	-1.0551338
51	H	6.1267999	-2.6860187	1.5378365
52	H	7.6371035	-4.5760256	2.055082
53	H	9.988069	-4.5610709	1.2399088
54	H	10.8014047	-2.6360363	-0.111252
55	H	9.2801943	-0.7607991	-0.6502308
56	H	8.4096625	0.4607617	1.9369191
57	H	9.2418914	2.7853944	2.0644122
58	H	8.5029229	4.4763063	0.3946573
59	H	6.9302597	3.8012714	-1.4132118
60	H	6.1182287	1.4710381	-1.5529008
61	H	2.7332144	1.7875298	1.678164
62	H	0.9780535	1.755561	1.6105075
63	H	1.02683	3.0161871	-0.557567
64	H	1.771513	3.9095957	0.7663733
65	H	3.2953779	2.3138933	-1.3635123
66	H	4.0429726	3.1905509	-0.0354142
67	H	2.3308088	4.465475	-2.2495427
68	H	4.0943577	4.5528474	-2.1354538
69	H	3.087836	5.3490057	-0.9164763
70	H	-7.4279534	0.872711	-1.8280826
71	H	-7.3394676	0.191189	-0.1859539
72	H	-7.0303258	3.1408168	-0.9443141
73	H	-6.5776318	2.4677467	0.6421063
74	H	-9.3516604	2.1907057	-0.6242115
75	H	-8.8661934	1.6340483	0.9758325
76	H	-10.0730933	3.8191221	1.1386236
77	H	-8.889601	4.5696449	0.0580492
78	H	-8.3932989	4.0082033	1.6620868
79	C	-8.5476324	-3.2786996	0.3345866
80	H	-9.546682	-3.199821	0.7523013
81	Br	-5.2540397	0.2760543	2.1947197
82	Cl	-8.633359	-2.8073464	-1.3864055
83	Cl	-7.4575569	-2.2092812	1.2416924
84	H	-8.1661702	-4.2940997	0.383759

Table S7 Crystal data and structure refinement parameters for **OD2** (CCDC 928592) and **KK2** (CCDC 2448145).

Compound	**OD2**	**KK2**
Molecular formula	$C_{37}H_{38}BrN_3$	$C_{25}H_{34}BrN_3$
Formula weight	616.64	456.46
Number of reflection used for unit cell determination (2θ range/°)	32139 (6.24–54.96)	16970 (5.05–55.75)
Temperature/K	293(2)	100
Crystal System	orthorhombic	monoclinic
Space group	$Pca2_1$	$P2_1/c$
a/Å	10.325(6)	9.2397(2)
b/Å	14.767(9)	16.1343(4)
c/Å	41.20(2)	15.0485(4)
α/°	90	90
β/°	90	93.457(2)
γ/°	90	90
V/Å^3	6282(6)	2239.29(9)
Z	8	4
D_c/g cm^{-3}	1.279	1.354
F(000)	2528	960
Radiation	Mo-Kα (λ = 0.71073 Å)	Mo-Kα (λ = 0.71073 Å)
Crystal size/mm^3	0.03×0.03×0.03	0.03×0.03×0.03
Range of induces *h*; *k* ;*l*	-13, 12; -19, 9; -52, 53	-12, 11; -21, 20; -19, 19
Reflections collected (unique)	12204	5329
Reflection observed with $I_0>2\sigma I_0$	7319	4425
Number of parameters	742	262
Final R indexes [$I_0>2\sigma I_0$]	R_1 = 0.0684, wR_2 = 0.1851	R_1 = 0.0341, wR_2 = 0.0830
Final R indexes [all data]	R_1 = 0.0933, wR_2 = 0.2059	R_1 = 0.0462, wR_2 = 0.0871
Goodness-of-fit on F^2	0.972	1.016
Max. Shift/Error in final cycle	0.00	0.00
Max. peak in final diff. map/e Å^{-3}	0.74	0.76
Min. peak in final diff. map/e Å^{-3}	-0.58	-0.31

References

[1] T. Enoki, K. Matsuo, J. Ohshita, Y. Ooyama, *Phys. Chem. Chem. Phys.* **2017**, *19*, 3565–3574.

[2] D. Šegan, R. D. Vukićević, S. Šegan, N. Sojic, O. Buriez, D. Manojlović, *Electrochim. Acta* **2011**, *56*, 9968–9972.
