# Supplementary material for: Elucidation of Organohalogenochromism (OHC) of D–A Pyridinium and D‐π‐A Pyridinium Dyes: Effect of Halogen Bond
Source: Chem Asian J. 2025 Jun 23;20(18):e00746. doi: 10.1002/asia.202500746 (PMC12450044; doi:10.1002/asia.202500746)

checkCIF/PLATON report

Structure factors have been supplied for datablock(s) KK2

THIS REPORT IS FOR GUIDANCE ONLY. IF USED AS PART OF A REVIEW PROCEDURE FOR PUBLICATION, IT SHOULD NOT REPLACE THE EXPERTISE OF AN EXPERIENCED CRYSTALLOGRAPHIC REFEREE.

No syntax errors found. CIF dictionary Interpreting this report

Datablock: KK2

Bond precision:	C-C = 0.0029 A		Wavelength=0.71073
Cell:	a=9.2397(2)	b=16.1343(4)	c=15.0485(4)
	alpha=90	beta=93.457(2)	gamma=90
Temperature:	100 K		

	Calculated	Reported
Volume	2239.29(10)	2239.29(9)
Space group	P 21/c	P 21/c
Hall group	-P 2ybc	-P 2ybc
Moiety formula	C25 H34 N3, Br	?
Sum formula	C25 H34 Br N3	C25 H34 Br N3
Mr	456.45	456.46
Dx,g cm-3	1.354	1.354
Z	4	4
Mu (mm-1)	1.852	1.852
F000	960.0	960.0
F000’	959.12	
h,k,lmax	12,21,19	12,21,19
Nref	5330	5329
Tmin,Tmax	0.946,0.946	0.874,1.000
Tmin’	0.946	

Correction method= # Reported T Limits: Tmin=0.874 Tmax=1.000
AbsCorr = MULTI-SCAN

Data completeness= 1.000 Theta(max)= 27.877

R(reflections)= 0.0341(4425) wR2(reflections)= 0.0871(5329)

S = 1.016 Npar= 262

The following ALERTS were generated. Each ALERT has the format
test-name_ALERT_alert-type_alert-level.
Click on the hyperlinks for more details of the test.

Alert level C

```
PLAT094_ALERT_2_C Ratio of Maximum / Minimum Residual Density ....       2.44 Report
```

Alert level G

```
PLAT883_ALERT_1_G Absent Datum for _atom_sites_solution_primary ..     Please Do !
PLAT899_ALERT_4_G SHELXL2018 is Outdated   and Succeeded by SHELXL     2019/3 Note
PLAT910_ALERT_3_G Missing # of FCF Reflection(s) Below Theta(Min).          2 Note
                1  0  0,   0  1  1,
PLAT941_ALERT_3_G Average HKL Measurement Multiplicity ..........        3.2 Low
PLAT965_ALERT_2_G The SHELXL WEIGHT Optimisation has not Converged     Please Check
PLAT969_ALERT_5_G The 'Henn et al.' R-Factor-gap value ..........      3.246 Note
              Predicted wR2: Based on SigI**2  2.68 or SHELX Weight  8.57
PLAT978_ALERT_2_G Number C-C Bonds with Positive Residual Density.          7 Info
```

0 **ALERT level A** = Most likely a serious problem - resolve or explain
0 **ALERT level B** = A potentially serious problem, consider carefully
1 **ALERT level C** = Check. Ensure it is not caused by an omission or oversight
7 **ALERT level G** = General information/check it is not something unexpected

1 ALERT type 1 CIF construction/syntax error, inconsistent or missing data
3 ALERT type 2 Indicator that the structure model may be wrong or deficient
2 ALERT type 3 Indicator that the structure quality may be low
1 ALERT type 4 Improvement, methodology, query or suggestion
1 ALERT type 5 Informative message, check

It is advisable to attempt to resolve as many as possible of the alerts in all categories. Often the minor alerts point to easily fixed oversights, errors and omissions in your CIF or refinement strategy, so attention to these fine details can be worthwhile. In order to resolve some of the more serious problems it may be necessary to carry out additional measurements or structure refinements. However, the purpose of your study may justify the reported deviations and the more serious of these should normally be commented upon in the discussion or experimental section of a paper or in the "special_details" fields of the CIF. checkCIF was carefully designed to identify outliers and unusual parameters, but every test has its limitations and alerts that are not important in a particular case may appear. Conversely, the absence of alerts does not guarantee there are no aspects of the results needing attention. It is up to the individual to critically assess their own results and, if necessary, seek expert advice.

Publication of your CIF in IUCr journals

A basic structural check has been run on your CIF. These basic checks will be run on all CIFs submitted for publication in IUCr journals (*Acta Crystallographica*, *Journal of Applied Crystallography*, *Journal of Synchrotron Radiation*); however, if you intend to submit to *Acta Crystallographica Section C* or *E* or *IUCrData*, you should make sure that full publication checks are run on the final version of your CIF prior to submission.

Publication of your CIF in other journals

Please refer to the *Notes for Authors* of the relevant journal for any special instructions relating to CIF submission.

PLATON version of 02/02/2025; check.def file version of 02/02/2025

Datablock KK2 - ellipsoid plot

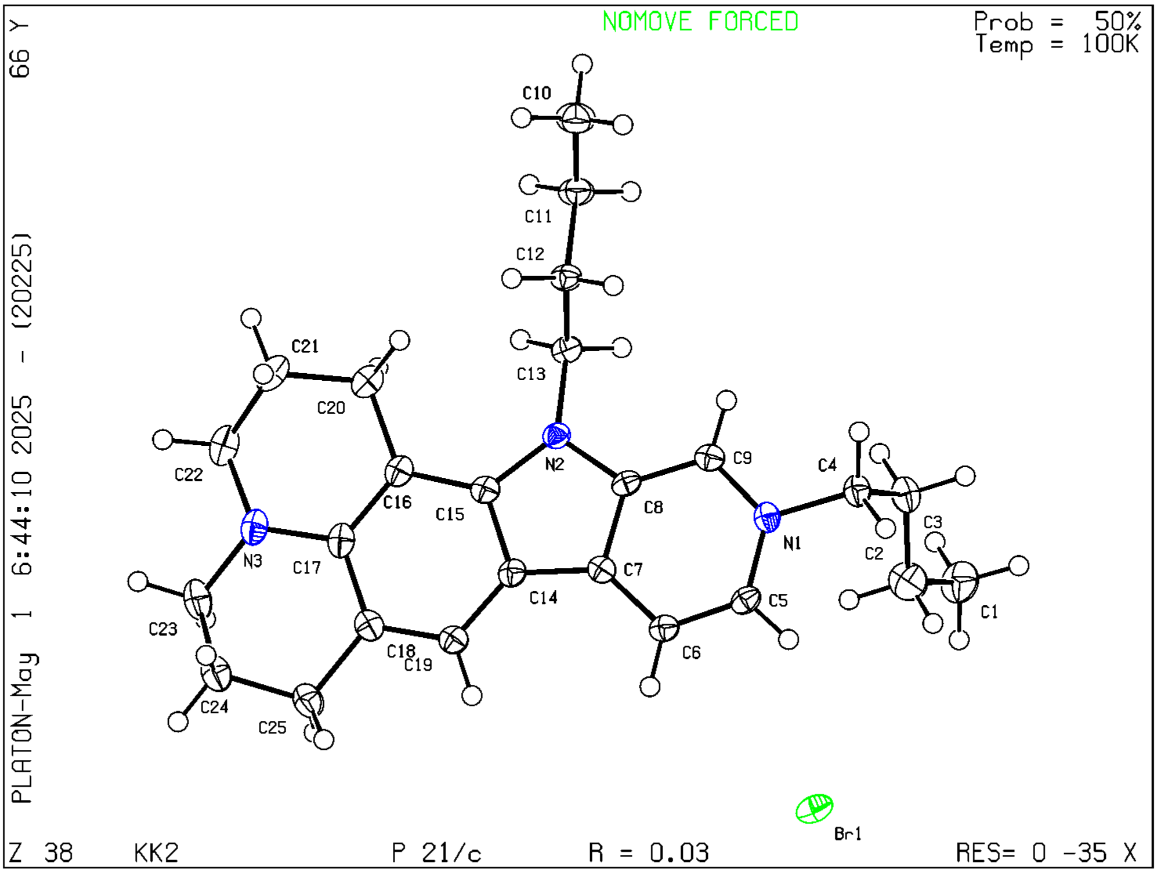

Supplement: Supplementary file 2 — Supporting Information [file ASIA-20-e00746-s001.zip › KK2_checkcif.pdf]
